# Intercropping Systems Modify Desert Plant-Associated Microbial Communities and Weaken Host Effects in a Hyper-Arid Desert

**DOI:** 10.3389/fmicb.2021.754453

**Published:** 2021-11-03

**Authors:** Zhihao Zhang, Xutian Chai, Akash Tariq, Fanjiang Zeng, Xiangyi Li, Corina Graciano

**Affiliations:** ^1^Xinjiang Key Laboratory of Desert Plant Roots Ecology and Vegetation Restoration, Xinjiang Institute of Ecology and Geography, Chinese Academy of Sciences, Urumqi, China; ^2^State Key Laboratory of Desert and Oasis Ecology, Xinjiang Institute of Ecology and Geography, Chinese Academy of Sciences, Urumqi, China; ^3^Cele National Station of Observation and Research for Desert-Grassland Ecosystem, Cele, China; ^4^University of Chinese Academy of Sciences, Beijing, China; ^5^Faculty of Agricultural and Forestry Sciences, Institute of Plant Physiology, National Council for Scientific and Technical Research, National University of La Plata, Buenos Aires, Argentina

**Keywords:** intercropping systems, co-occurrence networks, host effects, desert ecosystem, rhizosphere microbiomes

## Abstract

Intercropping is an important practice in promoting plant diversity and productivity. Compared to the accumulated understanding of the legume/non-legume crop intercrops, very little is known about the effect of this practice when applied to native species on soil microbial communities in the desert ecosystem. Therefore, in the present study, bulk soil and rhizosphere microbial communities in the 2-year *Alhagi sparsifolia* (legume)/*Karelinia caspica* (non-legume) monoculture vs. intercropping systems were characterized under field conditions. Our result revealed that plant species identities caused a significant effect on microbial community composition in monocultures but not in intercropping systems. Monoculture weakened the rhizosphere effect on fungal richness. The composition of bacterial and fungal communities (β-diversity) was significantly modified by intercropping, while bacterial richness (Chao1) was comparable between the two planting patterns. Network analysis revealed that Actinobacteria, α- and γ-proteobacteria dominated bulk soil and rhizosphere microbial co-occurrence networks in each planting pattern. Intercropping systems induced a more complex rhizosphere microbial community and a more modular and stable bulk soil microbial network. Keystone taxa prevailed in intercropping systems and were Actinobacteria-dominated. Overall, planting patterns and soil compartments, not plant identities, differentiated root-associated microbiomes. Intercropping can modify the co-occurrence patterns of bulk soil and rhizosphere microorganisms in desert ecosystems. These findings provided a potential strategy for us to manipulate desert soil microbial communities and optimize desert species allocation in vegetation sustainability.

## Introduction

Globally, drylands cover ∼45% of the earth’s total land area and are especially susceptible to climate change (Huang et al., 2015; Prăvălie, 2016). Plants growing in desert ecosystems have developed specific molecular and physiological responses that enable them to adapt to abiotic stresses such as long-term high radiation, low soil nutrition, severe soil salinity, and drought (Soussi et al., 2016). Root-associated microbiotas contribute to this adaptation (Mukhtar et al., 2021). A set of bulk soil microbiomes are recruited to the root vicinity (rhizosphere) by host plant root exudates, in which some plant growth promotion (PGP) microbial taxa in the rhizosphere can help their host plants absorb nutrients and enhance stress resistance and immunity (Rout and Southworth, 2013; Edwards et al., 2015; Vandenkoornhuyse et al., 2015; Mukhtar et al., 2021). On account of this intimate interrelationship, perturbations in the abiotic environment that affect either plants or their associated microbial communities are expected to also affect the other (Wardle et al., 2004), along with carbon (C), nitrogen (N), and phosphorus (P) biogeochemical cycles (de Vries et al., 2016; Averill et al., 2019; Schmidt et al., 2019). Many studies have reported that plant-microbe interactions are sensitive to plant species identities (e.g., legume/non-legume) and planting patterns (e.g., monoculture/intercrop) (Marschner et al., 2005; Koutika et al., 2007; Gong et al., 2019; Zhang et al., 2021). The symbiosis between microorganisms and plants drives vital ecological functions and service patterns associated with nutrient availability in soil ecosystems (Lekberg et al., 2013), highlighting the importance of microbial ecology research.

Intercropping, a classic strategy for maximizing plant diversity, is usually used in agroecosystems to suppress replant disease and improve crop productivity (Kumar et al., 2013). This practice generates a mosaic of niches for microorganisms and soil resources, thereby increasing underground biodiversity and improving the ability to restore its original function after interference (Kumar et al., 2013). Gong et al. (2019) found that intercropping improved rhizosphere soil fertility and enzymatic activity by modifying rhizosphere microbial communities, which significantly differed from those in monoculture systems. Appropriate intercropping of non-legume and legume crops is known to be beneficial and is practiced globally (Solanki et al., 2019). N-fixing bacteria can assimilate the atmospheric N, which, in turn, reduces and even avoids the need for N-fertilizer (Pelzer et al., 2012). More generally, the soil microbiome may contribute to the added value of intercropping systems, because different microbial species often interact with each other, forming a complex network (Agler et al., 2016; Hartman et al., 2017). The complexity of microbial networks promotes ecosystem multifunctionality related to nutrient cycling (Wagg et al., 2019). Therefore, it is necessary to comparatively investigate the diversity, composition, and co-occurrence patterns of soil microorganisms (bulk soil and rhizosphere) in monoculture or intercropping systems.

Currently, our knowledge on intercropping systems from a microbial perspective primarily comes from agroecosystems, with a limited understanding of the combinatorial effects of different indigenous species in desert ecosystems. Compared with agroecosystems in the oasis, water and nutrients in virgin desert soil are relatively scarce. Plant growth and distribution are limited by the spatiotemporal availability of water and nutrients in arid ecosystems (Zeng et al., 2006). However, these situations can be improved by the hydraulic redistribution of phreatophyte species (Hultine et al., 2004). For example, in the hyper-arid Taklamakan Desert, northwestern China, the herbaceous perennial legume *Alhagi sparsifolia* can transfer water and nutrients to the shallow soil by its deep-rooted systems and symbiotic microbiota (Zeng et al., 2006), providing resources for neighboring plants. *Karelinia caspica* is a salinity- and drought-tolerant herbaceous perennial species, with a relatively shallow root system. These plants dominate in this region and are often used as pioneer sand-fixing plants and forage grasses. Theoretically, the intercropping of these two species with different rooting depths can perpendicularly maximize soil space utilization, introduce more microbial niches in desert soil, and achieve greater C and N status in plant and soil compared to monoculture based on available evidences (Sayyad et al., 2006; Du et al., 2019), but this needs to be explored further.

In the present study, we investigated the effect of *A. sparsifolia/K. caspica* monoculture vs. intercropping on bulk soil and rhizosphere microbes. The biodiversity, structure, and co-occurrence networks of microbial communities were characterized by high-throughput sequencing of 16S rRNA and Internal Transcribed Spacer (ITS) genes in monoculture and intercropping systems. These attempts are intended to provide new ideas for soil fertility improvement and maintenance of desert vegetation.

## Materials and Methods

### Study Sites and Experimental Design

We conducted our study at Cele National Station of Observation and Research for Desert-Grassland Ecosystem (80°43′45″E, 37°00′57″N), located at the periphery of Cele Oasis on the southern edge of the Taklamakan Desert, Southern Xinjiang, China. The study site was characterized by a hyper-arid climate, with mean annual precipitation <50 mm, a mean annual potential evaporation of 2,595 mm, and a mean annual temperature of 11.9°C (Gui et al., 2013). The soil in this region is classified as Aridisol according to the United States Department of Agriculture, Soil Taxonomy (USDA ST) system.

Two planting patterns, *A. sparsifolia* and *K. caspica* monoculture and their intercrop, were set up from July 2017 to September 2018 under field conditions. *A. sparsifolia* and *K. caspica* seedlings were established by seeds and tubers, respectively, according to the conventional seedling raising method. To avoid differences in growth rates, *K. caspica* was planted 1 year after the planting of the *A. sparsifolia* seedlings. Specifically, *A. sparsifolia* seeds were collected from the natural desert area in 2016 and were sown in seedling cups in May 2017. After 1 month, uniform seedlings were transplanted to the experimental plots. *K. caspica* seedlings were bred in plots by tuberous roots excavated from the nearby desert area in March 2018. These two species were arranged in two patterns: the monoculture of *A. sparsifolia* and *K. caspica* and their intercrop (Supplementary Figure 1). Buffers were set between each plot. Each pattern had three duplicate plots. All plots got unified management and adequate irrigation during the growing season (from April to September) before 2020, after which the plants grew naturally except for the removal of weeds.

### Sampling Collection

The roots of both plants coexisted in a layer of 30–50 cm. We collected bulk and rhizosphere soil using a root auger in this range. Soil that was not attached to roots and could be shaken off was collected as the bulk soil. Next, we put the roots into a 50-mL sterile tube and brought them back to the laboratory in an icebox. The soil that had adhered to the root surface was shaken down with a vortex oscillator to collect the rhizosphere soil. In total, 24 samples were collected [2 plant species × 2 planting patterns (monoculture and intercrop) × 2 soil compartments (bulk soil and rhizosphere) × 3 replicates] and stored at −80°C until DNA extraction. Three soil cores were collected from the layer of 30–50 cm in each plot between plant rows to check the soil properties (Supplementary Table 1).

### Soil Properties Analysis

The K_2_Cr_2_O_7_-H_2_SO_4_ oxidation method was applied to determine dissolved organic carbon (DOC) and soil organic matter (SOM) concentration (Nelson et al., 1982). Total N in soil was measured after digestion with H_2_SO_4_-H_3_BO_3_ (He et al., 1990). Soil total P was determined using the alkali fusion-Mo-Sb Anti spectrophotometric method (Chen et al., 2018). After soaking in HF–HNO_3_–H_2_O_2_ overnight, soil samples were digested and evaluated for total K using inductively coupled plasma optical emission spectrometry (iCAP 6300, Thermo Fisher Scientific, Waltham, MA, United States). Soil moisture content was the weight lost after drying for 24 h at 105°C. Soil pH was measured with a pH meter (PHBJ-260, INESA Scientific Instrument Co., Ltd., Shanghai, China) in a 1:2.5 soil: CaCl_2_ solution. Soil electrical conductivity (EC) was determined by a conductivity meter (YD28; INESA Scientific Instrument Co., Ltd., Shanghai, China).

### DNA Extraction, Amplicon, and Sequencing

Total soil genomic DNA was extracted using a DNeasy PowerSoil DNA isolation kit (Qiagen, Inc., Düsseldorf, Netherlands) from 0.5 g of bulk soil and rhizosphere soil according to the instructions. The DNA concentrations were determined using a Qubit Fluorometer (Invitrogen, United States), and the quantity and quality of DNA extracted were checked using a NanoDrop ND-1000 spectrophotometer (Thermo Fisher Scientific, Waltham, MA, United States) and agarose gel electrophoresis, respectively. The V3-V4 hypervariable regions of the bacterial 16S rRNA gene were amplified using the PCR primer pair 338F/806R (Claesson et al., 2009). A library of fungal amplified ITS1 regions was generated using the PCR primer pair ITS5/ITS2 (White et al., 1990). Error-tolerant 7 bp barcodes were integrated into the primers for multiple sequencing. The PCR reaction mixtures—0.25 μL Q5 High-Fidelity DNA Polymerase (5U/μL), 5.0 μL Q5 Reaction Buffer (5×), 1.0 μL of each primer, 5.0 μL High-Fidelity GC Buffer (5×), 2.0 μL dNTPs (2.5 mM), 2.0 μL template DNA, and 8.75 μL ddH_2_O—were amplified using thermocycling method according to the following conditions: 2 min (for 16S)/30 s (for ITS) at 98°C for initial denaturation, followed by 30 cycles of 15 s for denaturation at 98°C, 30 s for annealing at 50°C, 30 s for the extension at 72°C, and then 5 min for a final extension at 72°C. The sequencing libraries were generated by a TruSeq DNA Sample Preparation Kit (Illumina, San Diego, United States) and a Template Prep Kit (Pacific Biosciences, Menlo Park, United States). High-throughput sequencing of purified PCR amplicons was then performed using the Illumina MiSeq platform (Personal Biotechnology Company, Shanghai, China).

### Bioinformatics Analysis

Raw reads were analyzed using a QIIME2 (version 2019.4) bioinformatics platform. The primer fragments and unmatched primer sequences of each library were removed by executing the *qiime cutadapt trim-paired* command. Quality filtering, denoising, splicing, and chimera removal were performed on each library using the *qiime dada2 denoise-paired* method. Differing from the VSEARCH method with 97% similarity clustering, the DADA2 method selected in this paper only carried out de-duplication, which was equivalent to 100% similarity clustering (Callahan et al., 2016). Each sequence that was qualitatively controlled by the DADA2 method was referred to as an amplicon sequence variant (ASV). ASVs with a total number of sequences of 1 in all samples (singleton ASV) were eliminated, and then the ASVs were merged. After removing the sequences relating to chloroplasts (60 sequences) and mitochondria (4 sequences), the high-quality sequences from all the samples were subsequently studied. The bacterial 16S rRNA and fungal ITS sequences were annotated in the Silva database (Release 132) (Quast et al., 2013) and UNITE database (Release 8.0) (Abarenkov et al., 2010), respectively. For each ASV, the classify-sklearn algorithm implemented in QIIME2 was used to perform Naïve Bayes species annotation. To reduce the difference in the size of pools among different samples, the subsequent analysis was carried out at the same sequencing depth. We adopted the rarefaction method to randomly extract a certain number of sequences from each sample pool to reach the same depth, to predict the number and abundance of ASVs observed by each sample at that sequencing depth.

### Statistical Analysis

All statistical analyses were performed using QIIME2 and R software (version 4.0.4) (R Core Team, 2020). The *Shapiro test* function was used to test the normality of the data and the *bartlett.test* function in the multcomp package was used to test the homogeneity of the variance of the data (Torsten et al., 2008). Intergroup comparison of each parameter was divided into two cases for analysis. If the data conformed to normal distribution and homogeneity of variance, the *aov* function in the R package *stats* was used for the analysis of variance (ANOVA). If the differences from groups were statistically significant, the least significant difference (LSD) method (*LSD.test* function) was used post-test for multiple comparisons among groups. If the data was not normal or the variance was not uniform, a Kruskal–Wallis non-parametric test method was adopted. If the differences between groups were statistically significant, the *kruskal.test* function was used to compare the mean from multiple groups. The R package *ggplot2* was used to generate all figures (Wickham, 2016).

#### Microbial Taxonomic Composition

We visualized the relative abundance of microbial dominant taxa (>1% relative abundance) at the phylum level (class level for phylum Proteobacteria) and investigated the effects of plant type, planting pattern, and soil compartment on their abundances.

#### Alpha Diversity

The unfiltered ASV table was used to calculate the α-diversity, which was the average score of the Chao1 index at the maximum flattening depth (95% of the sample sequence size at the lowest sequencing depth among all the samples) calculated by the *qiime diversity alpha-rarefaction* command implemented in QIIME2. We tested the effects of plant type, planting patterns and soil compartments on bacterial and fungal Chao1 indexes.

#### Beta Diversity

We conducted a permutational analysis of variance (PERMANOVA) to quantify the effects of plant species, planting patterns, and soil compartments on microbial communities (Bray–Curtis dissimilarities) individually using the functions *adonis* implemented in the R package *vegan* with 999 permutations. Next, an unconstrained principal coordinate analysis (PCoA) was performed to visualize the effects of major factors on bacterial and fungal community composition. The filtered ASV sequence counts were normalized using the TMM (trimmed mean of *M*-values) method implemented in the R package *edgeR* (Robinson et al., 2010), and then they were expressed as relative abundance counts per million (CPM) for different sets of the microbial kingdom and planting patterns. To conduct an in-depth analysis of microbial data in bulk soil and rhizosphere communities, the threshold of microbial sequence counts was set for the normalized ASV table: ASV contained two sequences (avoiding single-count ASV) and occurred in over three samples (replicates per treatment). ASVs remaining after this filtration step comprised the bulk soil and rhizosphere microbial communities.

#### Planting-Pattern-Sensitive ASVs Identification

The ASV point-biserial correlation coefficient (r), which was positively correlated with monoculture or intercrop patterns, was calculated using indicator species analysis based on correlation with 999 permutations and considered significant at *P* < 0.05 with the R package *indicspecies* (de Cáceres et al., 2010). The likelihood ratio tests implemented in the R package *edgeR* were also used to test the differential ASV abundance between the two planting patterns. ASVs that differed in abundance between monoculture and intercrop patterns were identified with *P* < 0.05 corrected by false discovery rate (FDR), and these ASVs were considered to have planting-pattern sensitivity. ASVs confirmed by both indicator species analysis and likelihood ratio test were defined as Planting-Pattern-Sensitive ASVs (*ps*ASVs).

#### Co-occurrence Network Constructions and Analysis

The co-occurrence network can characterize microbial-microbial interactions, which helps us understand the effects of planting patterns and soil compartments on interactions among microorganisms. To diminish sporadic genera in the data set, we selected the genera that were present in all samples and their relative abundance was more than 0.01%. Robust correlations (ρ > 0.7, *P* < 0.01) calculated with the R package *psych* were used to construct the individual networks. The FDR method implemented in the package *psych* was used to adjusted all *P*-values. In co-occurrence networks, each node represented a genus, and each edge connected two nodes represented a strong and significant correlation. We described the network topological properties with the R package *igraph* (Csardi and Nepusz, 2006), including the number of nodes and edges, the density of edge, diameter, average path length, average clustering coefficient, average degrees, and modularity calculated with the greedy modularity algorithm (Clauset et al., 2004). We used the interactive platform Gephi to visualize these co-occurrence networks (Bastian et al., 2009). Additionally, the bulk soil and rhizosphere meta-networks were visualized at the ASV level used the Fruchterman-Reingold layout with 10,000 permutations implemented in the package *igraph*, which help find keystone ASVs sensitive to both planting patterns. Nodes ranked in the top 1% based on the number of degrees were defined as keystone taxa.

## Results

### Bulk Soil and Rhizosphere Microbiota

We profiled bacterial and fungal communities from 12 bulk soil and 12 desert plant rhizosphere samples under two planting systems (Supplementary Figure 1) to investigate the effects of legume (*A. sparsifolia*) and non-legume (*K. caspica*) monoculture and their intercropping systems on microbial communities in desert soil. The bacterial community profiling yielded a total of 1,760,559 high-quality sequences (range 53,772∼93,979, median 73,202.5). The fungal community profiling yielded 1,892,987 high-quality sequences (range 60,911∼92,172, median 80,099.5). We identified 111,425 bacterial and 7,394 fungal ASVs across all the samples (Supplementary Figure 2). An average of 62.63% of bacterial ASVs was annotated to the genus level. For fungi, 30% and 34% of ASVs were annotated at species and domain level, respectively. The amount of ASVs in bulk soil was higher than that in the rhizosphere except for fungal ASV in monoculture *K. caspica* rhizosphere (Supplementary Figure 2).

**FIGURE 1:**
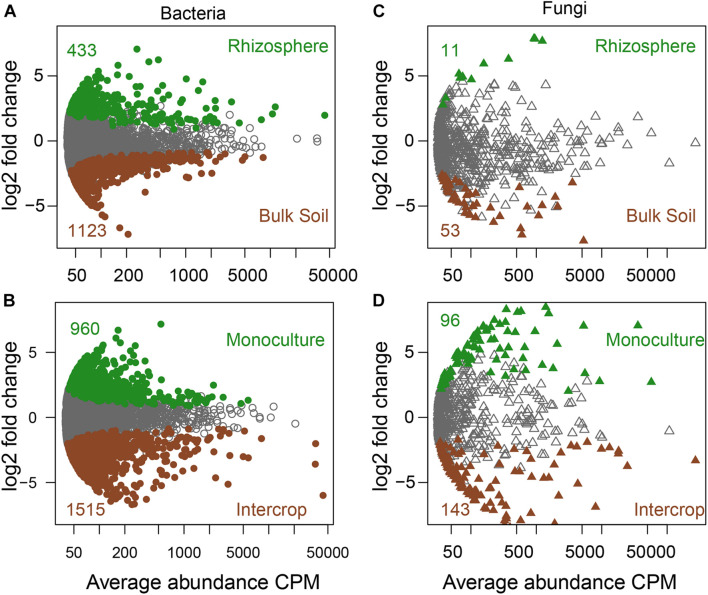
The specific sets of microbes in bulk soil and rhizosphere **(A,C)** and different planting patterns **(B,D)**. MA plots showing the abundance patterns of bacteria and fungi in different soil compartments (bulk soil/rhizosphere) and planting patterns (monoculture/intercrop). *X*-axis display average ASV abundance as counts per million (CMP), and *Y*-axis report the log2-fold change (rhizosphere related to bulk soil, monoculture relate to intercrop). ASVs with significant differences were labeled in color (green or brown), while those without significant differences were labeled in gray (likelihood ratio test, *P* < 0.05, FDR corrected).

**FIGURE 2:**
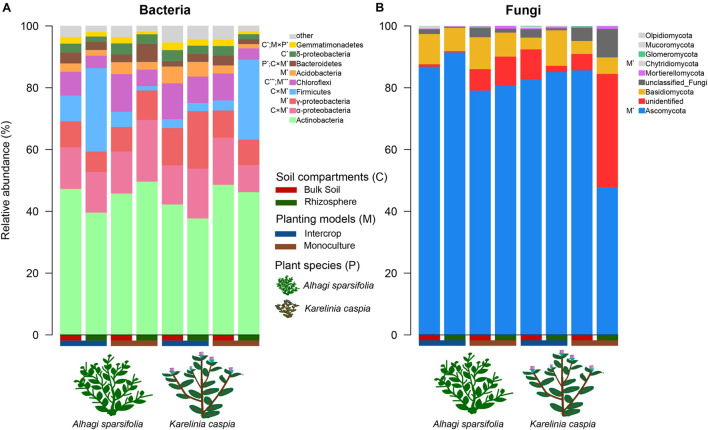
The relative abundance of dominant (>1%) phyla in bacterial **(A)** and fungal communities **(B)**. ANOVA was used to quantify the effects of plant species (P), planting models (M), and soil compartments (C) on the relative abundance of dominant bacterial and fungal groups, which are shown in the legend. The multiple signs denote interactions. *P < 0.05, ***P < 0.001.

Soil compartments (bulk soil vs. rhizosphere) and planting patterns (intercrop vs. monoculture) harbored different sets of microbial ASVs (Figure 1). The composition of dominant species (relative abundance >1%) was shown in Figure 2. The relative abundances of Actinobacteria, Basidiomycota, Mortierellomycota, Glomeromycota, Mucoromycota, and Olpidiomycota were slightly affected by planting patterns, soil compartments, and host species types (*P* > 0.05). Yet host species types only markedly impacted the relative abundance of Bacteroidetes and Gemmatimonadetes. Planting pattern was the main factor affecting other dominant phyla, such as Alphaproteobacteria (α-proteobacteria), Gammaproteobacteria (γ-proteobacteria), Firmicutes, Chloroflexi, Acidobacteria, Bacteroidetes, Gemmatimonadetes, Ascomycota, and Chytridiomycota. The changes of these dominant phyla abundance possibly influenced the β-diversity of microbial communities where they were present.

**FIGURE 3:**
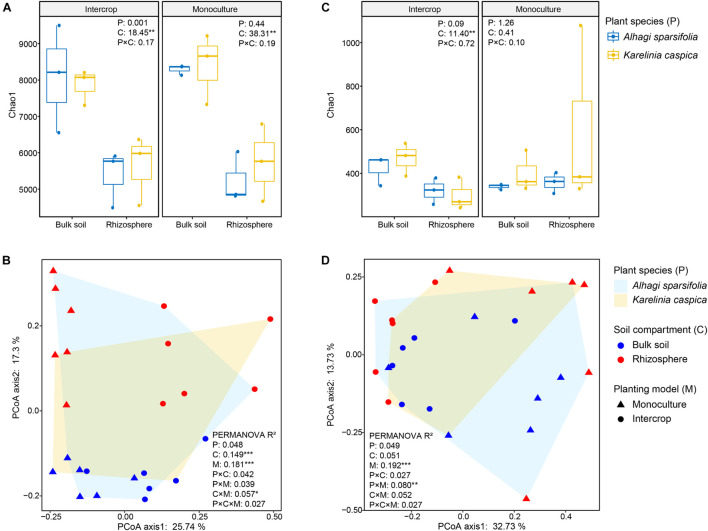
Bacterial and fungal diversity measurements of different plant species (P), planting models (M), and soil compartments (C). **(A,C)** Show the Chao1 index of bacterial and fungal communities of *A. sparsifolia* and *K. caspica* in monoculture and intercropping systems, respectively. Different letters indicate significant differences according to the LSD test or Kruskal–Wallis test. The multiple signs denote interactions. *P < 0.05, **P < 0.01, ***P < 0.001. **(B,D)** Show community composition (based on ASV Bray-Curtis distance) performed by PCoA, respectively. PERMANOVA with 999 permutations tested the effect (*R*^2^) of planting patterns (M) and soil compartments (C) and their interaction (M × C) on bacterial and fungal communities. *P < 0.05, **P < 0.01, ***P < 0.001.

We rarified the microbial communities to a certain depth (95% of the minimum number of sequences, 51,083 sequences for bacteria, and 57,865 sequences for fungi) to calculate α-diversity (indicated by Chao1), and evaluated β-diversity (the difference between microbial communities) by Bray–Curtis distance matrix (Figure 3). Soil compartments independently significantly affected the richness of bacterial communities in both planting patterns, while fungal richness was only influenced in intercropping systems. The richness of microbial community in bulk soil was higher in both planting patterns than in rhizosphere except for fungal community in monoculture pattern (*P* > 0.05, Figure 3C). The PERMANOVA was used to quantify the effect of plant species, planting patterns, and soil compartments on the β-diversity of bacterial and fungal communities (Table 1), which was visualized by PCoA (Figures 3B,D). The genetic background of plant species had no significant effect on microbial β-diversity in intercropping systems, while significantly affected it in monoculture (Table 1). Planting patterns (*R*^2^ = 0.181, *P* < 0.001), soil compartments (*R*^2^ = 0.149, *P* < 0.001), and their interactions (*R*^2^ = 0.057, *P* < 0.05) exerted a significant impact on the β-diversity of the bacterial communities. However, the β-diversity of fungal communities were changed by planting patterns (*R*^2^ = 0.192, *P* < 0.001) and their interaction with plant types (*R*^2^ = 0.080, *P* < 0.01).

**TABLE 1:** The results of PERMANOVA quantifying the effects of plant species and soil compartments on bacterial and fungal communities in each planting pattern.

	Intercrop				Monoculture			
	Bacteria		Fungi		Bacteria		Fungi	
	*R* ^2^	*P*	*R* ^2^	*P*	*R* ^2^	*P*	*R* ^2^	*P*
Plant species (P)	0.082	0.321	0.143	0.063	**0.130**	**0.032**	**0.167**	**0.018**
Soil compartment (C)	**0.244**	**0.006**	**0.157**	**0.042**	**0.260**	**0.001**	0.115	0.110
P × C	0.068	0.475	0.032	0.993	0.093	0.142	0.081	0.431

*Significant effects are indicated in bold.*

### Planting-Pattern-Sensitive Amplicon Sequence Variants

Indicator species analysis was employed to identify individual bacterial and fungal ASVs in bulk soil and rhizosphere communities whose abundances varied between the intercropping and monoculture patterns (Supplementary Table 2 and Supplementary Data 1). Of the bacterial and fungal ASVs in the bulk soil communities, 18.65%, and 13.79%, respectively, positively responded to the planting patterns. In the rhizosphere communities, 11.45% and 20.11%, respectively, of the bacterial and fungal ASVs were sensitive to the planting patterns. More bacterial and fungal ASVs responded to the intercropping system than monoculture.

We validated these indicator ASVs by the likelihood ratio test in the R package *edgeR* (Supplementary Data 2). Next, we assessed the ASVs that were filtered by both methods as planting-pattern-sensitive ASVs (hereafter *ps*ASVs; Supplementary Data 3). There were 233 and 7 bacterial and fungal *ps*ASVs, respectively, in the bulk soil communities (Supplementary Figure 3), accounting for 8.81% and 1.25% of the total bulk soil community sequences. In the rhizosphere, we screened out 301 and 17 bacterial and fungal *ps*ASVs, respectively, accounting for 38.46% and 22.06% of the total rhizosphere community sequences. These proportions can be an approximation for an “effect size” of planting patterns on microbial communities. We observed 49 and 2 bacterial and fungal *ps*ASVs in the bulk soil and rhizosphere communities, respectively. These *ps*ASVs exhibited particularly taxonomic patterns with the associated planting patterns (Supplementary Figures 4, 5 and Supplementary Data 1, 2). Therefore, intercropping and monoculture patterns harbored a quite specialized subset of bulk soil and rhizosphere microbiota.

### Effects of Planting Patterns on Microbial Co-occurrence Patterns

**FIGURE 4:**
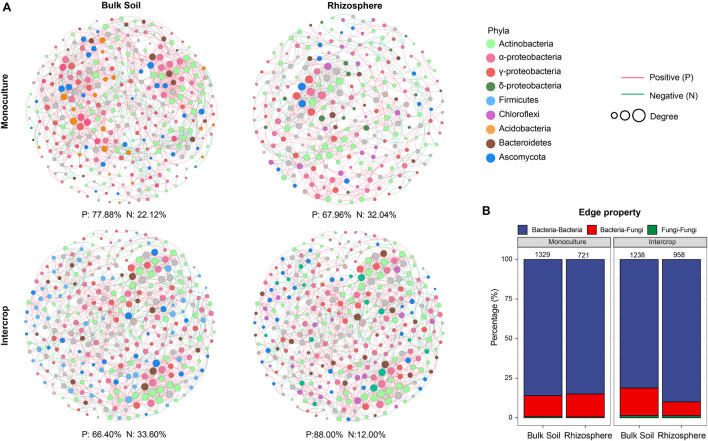
The microbial co-occurrence network of bulk soil and rhizosphere in each planting pattern. The nodes (individual circles) represent genera in the data sets, and their sizes are proportional to a degree. Network edges stand for strong (Spearman’s ρ was >0.7) and significant (FDR-adjusted *P*-value was <0.01) correlation. Green and red lines represent positive and negative correlations, respectively. **(A)** Individual networks; **(B)** edge property. The numbers of edges show above bars. Other properties of the network can be found in Table 2.

The co-occurrence pattern among microbiota is an important issue in the study of microbial ecology and can be visualized by network analysis. The separate co-occurrence networks for bulk soil and rhizosphere microbial communities were illustrated in Figure 4A. Actinobacteria, α-proteobacteria, and γ-proteobacteria dominated these co-occurrence networks. The interactions between microorganisms were mainly positive association, especially the rhizosphere network in intercropping systems. The proportion of inter-kingdom interactions was the highest in microbial networks of bulk soil in intercropping systems. The bulk soil microbial networks had more significant nodes (genera) than those in the rhizosphere network. Although the fewest genera were observed in the rhizosphere of intercropping systems, they displayed higher average degree, edge density, and average clustering coefficient (Table 2). The rhizosphere microbial network in monoculture had the largest diameter and average path length. Generally, microbial networks in intercropping were more modular than those in monoculture.

**TABLE 2:** The properties of microbial co-occurrence networks (Figure 4) in each planting pattern and soil compartments.

Planting patterns	Soil compartments	Number	Average degree	Edge density	Diameter	Average path length	Average clustering coefficient	Modularity
Monoculture	Bulk Soil	321	8.280	0.0259	17	6.020	0.471	0.642
	Rhizosphere	250	5.768	0.0232	23	7.098	0.446	0.697
Intercrop	Bulk Soil	329	7.526	0.0229	18	6.277	0.496	0.701
	Rhizosphere	242	7.917	0.0329	20	6.531	0.579	0.690

**FIGURE 5:**
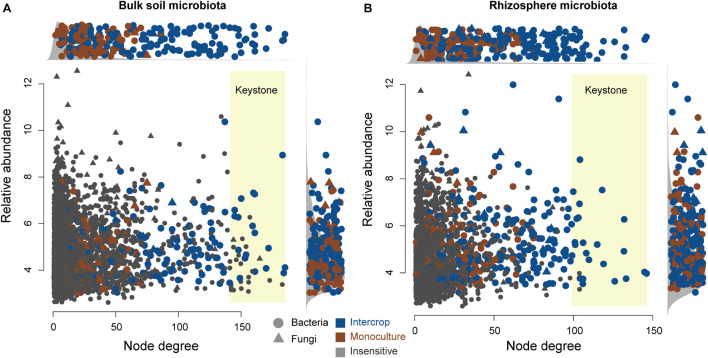
Degree and relative abundance of ASVs in bulk soil **(A)** and rhizosphere **(B)** microbial co-occurrence networks (Supplementary Figure 6). The relative abundance was calculated as counts per million (CMP). The circles and triangles represent bacteria and fungi, respectively. The blue and brown nodes represent nodes sensitive to intercrop and monoculture patterns, respectively. Gray nodes are insensitive to planting patterns. The keystone ASVs (top 1% degree of co-occurrence) have a yellow background. Side panels show the distributions of degree and abundance of ASVs sensitive to planting patterns.

Figure 5 shows the co-occurring degree and abundance of *ps*ASVs. In the bulk soil and rhizosphere communities, *ps*ASVs specific to the intercropping system exhibited a medium-to-high range of node degrees, while monoculture *ps*ASVs exhibited lower degrees of nodes. More significantly correlated nodes, connections, and keystone nodes and higher network connectivity (node degrees) occurred in bulk soil than in the rhizosphere, which contained more *ps*ASVs (Table 2). Most keystone *ps*ASVs in the bulk soil community were from Actinobacteria, Bacteroidetes, Proteobacteria, and Ascomycota (Supplementary Data 5). In the rhizosphere community, Actinobacteria and Firmicutes comprised a major part of the keystone taxa.

## Discussion

In agricultural ecosystems, there have been many studies on the effects of different planting patterns (monoculture vs. intercropping systems) and crop species (leguminous and others) on root-associated microbial communities (Hartman et al., 2017; Solanki et al., 2020; Liu et al., 2021). However, in desert ecosystems, with its extremely limited water and nutrient availabilities, there has been a lack of attention to the effects of native dominant plant species allocation on soil microbial communities, which limits our understanding of the role of plant interaction in the underground ecological process from a microbial perspective.

### Intercropping Systems Weaken Host Effects on Root-Associated Microbial Communities

This study revealed a limited influence of plant species on microbial community structure in intercropping systems (Figure 3). We observed similar bulk soil and rhizosphere microbial communities between the two desert plants intercropping systems, while plant species identity exerted a significant effect on microbial community composition in monoculture. Plants can actively modify their root exudate property to recruit their specific rhizosphere microbial communities (Badri and Vivanco, 2009; Zhalnina et al., 2018). A recent study has shown that plants with different genetic backgrounds have a distinct composition of root-associated microbiomes (Fitzpatrick et al., 2018). Studies on 8 Arabidopsis ecotypes have also found host genotype-dependent root-associated microbial community (Lundberg et al., 2012). The effects of plant genotypes on microbial communities in monoculture systems in this study were similar to those in these pot experiments (Lundberg et al., 2012; Fitzpatrick et al., 2018), thus we conjectured that interactions between different plants in the intercropping system may have weakened their respective host effects. Possible explanations are as follows. Firstly, rhizosphere microorganisms are a subset of bulk soil microbiomes (Bulgarelli et al., 2012). Water-deficit can restrict the mobility of elements from dehydrated soil and reduce the sensitivity of soil nutrients to environmental changes (Gonzalez-Dugo et al., 2012). Although the physicochemical properties of soil under monoculture of two plants (e.g., C and N states; Supplementary Table 1) were slightly different, this difference still alters the bulk soil microbial community (the seed bank of rhizosphere microbial communities) (Vandenkoornhuyse et al., 2015). In intercropping systems, the same soil status and composition of litter provided similar microhabitat for microbial communities of these two plants. Secondly, the assembly of root-associated microbial community is driven by the dynamic exudation of root exudates and the preference of microorganisms for these exudates (Zhalnina et al., 2018). Root entanglement possibly promotes the interaction between the microbiome of the two plants, which may increase the comparability of the rhizosphere microbial communities of the two plants.

Soil compartments separated the rhizosphere bacterial community (α- and β- diversity) from the bulk soil under either planting pattern. Previous studies concerning other plant microbiomes also confirmed this partition (Santhanam et al., 2015; Dombrowski et al., 2017; Fan et al., 2017; Liu et al., 2020). The rhizosphere is a highly dynamic environment that is the primary site of plant nutrient and other metabolite output, providing a rich source of C and energy for nearby microorganisms (Dakora and Phillips, 2002; Bulgarelli et al., 2013). The gradient distribution of these resources on the soil-plant continuum differentiates the rhizosphere microbial community from the bulk soil (Schreiter et al., 2014; Chen et al., 2019). The richness of the bacterial community was affected only by soil compartments, while the community composition was affected by the interaction between soil compartments and planting patterns, implying that intercropping systems affect bacterial communities by changing their composition rather than their richness. However, fungal communities responded differently to planting patterns and soil compartments than bacteria did (Figures 1–3). The rhizosphere of two plant species in monoculture had a similar fungal community (α- and β-diversity) (Figures 3B,D and Table 1), which is similar with the observation for *Araucaria bidwillii* a tropical montane rainforest (Curlevski et al., 2010). While there is less information on arbuscular mycorrhizal (AM, belonging to the phylum Glomeromycota) fungus in this study, the presence of mycelium makes a specific fungal community unlikely to be restricted to the rhizosphere (Richard, 2005). Changes in planting patterns may potentially alter filamentous growth form. Sequencing of Glomeromycota requires the use of specific primers and protocols, which is likely the reason of the low recovery of this phylum in this study (Figure 2B). Next, the specialized methods or high-resolution methods (i.e., metagenomics) will be applicated to characterize the abundance and diversity of Glomeromycota, as well as their ecological processes in both planting patterns. Taken together, these findings demonstrate that microbiota from intercropping and monoculture differ significantly, and compartment-specific differences are more pronounced in bacteria than in fungi (Gong et al., 2019).

Our results also revealed a set of ASVs sensitive to planting patterns in both bulk soil and rhizosphere microbial communities (Supplementary Figures 3–5), and these microorganisms served as indicator species to explain the effect of planting patterns on the β-diversity of microbes in different soil compartments (Figure 3). For example, members of Firmicutes are salt-tolerant and nitrogen-fixing, which can promote plant growth (Mukhtar et al., 2017, 2021; Solanki et al., 2020). In the rhizosphere, there was a higher abundance of *ps*ASVs from Firmicutes in intercropping systems than monoculture, but similar results were not observed in bulk soil (Supplementary Figure 4), indicating rhizosphere microbes may account for the added value of intercropping systems by recruiting more beneficial microbial communities into the rhizosphere (Pivato et al., 2021). The association between changes in Firmicutes to the monoculture/intercropping system was found in earlier studies (Zhang M. M. et al., 2018; Gong et al., 2019; Solanki et al., 2020).

Although we found ASVs that are sensitive to cropping systems, it is difficult to infer their ecological function from taxonomic information alone (Langille et al., 2013). Other approaches, such as metagenome sequencing or culture-dependent methods, are needed to further determine how these sensitive microbes influence plant performance (Schlaeppi and Bulgarelli, 2015).

### Planting Pattern and Soil Compartment Changed Microbial Co-occurrence Patterns

The α-diversity of bacterial communities in the intercropping system did not differ markedly from that in the monoculture (Figure 3A), which is similar to the findings of previous studies on wheat-faba bean and wheat-pea monoculture/intercropping systems (Tang et al., 2016; Pivato et al., 2021), suggesting a conservative response of bacterial richness to planting patterns, that is, bacterial richness is independent of their neighboring plants. This is consistent with the view of Tkacz et al. (2020), who found that microbial communities are more affected by roots than by soil or plant species. However, the similarity of bacterial α-diversity between intercropping and monoculture systems may also be attributed to the characterization methods of microbial communities. These methods only provide information on the taxonomic composition and diversity; they do not provide information on the interactions between microbiomes or their functions (Pivato et al., 2021). Thus, we compared the microbial co-occurrence patterns of bulk soil and rhizosphere communities in intercropping and monoculture systems (Figure 4 and Supplementary Figure 6).

Network analysis can uncover non-random covariation patterns at the community level, which provides an approach for understanding microbial communities from an ecological perspective (Shi et al., 2016). We explored possible co-occurrence links among microbes in rhizosphere and bulk soil under each planting pattern and revealed that intercropping system and soil compartments can alter the microbial co-occurrence patterns. Networks with small path lengths are considered small-world networks and are associated with rapid responses of ecosystems to disturbances (Watts and Strogatz, 1998; Zhou et al., 2010). Therefore, compared with the bulk soil microbial community, these desert plant rhizosphere microbial communities may be insensitive to environmental changes. The edge density and average degree represent the complexity of the network (Shi et al., 2016; Zhang B. et al., 2018). In the present study, rhizosphere microbial network in intercropping systems presented higher edge density and average degree, that is, a larger proportion of actual interactions among microbes out of all possible links, which provoked a more complex system in the rhizosphere by intercropping. Since connections in co-occurrence networks may indicate ecological interactions or niche-sharing among microbes (Berry and Widder, 2014), intercropping systems may promote greater interactions or form more shared niches. Alternatively, more complex networks could also reflect the active state of many microbes in the rhizosphere in intercropping systems (Fierer and Lennon, 2011). A module is defined as a group of nodes that are highly covariant within the group with a few links outside sets (Armbruster et al., 2014), and it can stabilize networks by limiting external perturbation to a module. Intercrop increased the modularity of microbial network in bulk soil (Table 2), implying improved habitat heterogeneity and cluster of closely related species in bulk soil (Olesen et al., 2007). Furthermore, there is increasing evidence that ecological network features representing interactions between co-occurring organisms can influence the response of the microbial community to environmental changes (e.g., extreme climate events) (de Vries and Shade, 2013; de Vries and Wallenstein, 2017). The increased proportion of negative interaction between microorganisms in bulk soil under intercropping could improve the stability of the microbial network under the case of environmental disturbance, while increased positive interactions in the rhizosphere would reduce the stability of the network, resulting in positive feedback and the co-oscillation response of microorganisms to environmental fluctuations (Coyte et al., 2015). The lignin and cellulose in the soil are mainly degraded by fungi, and bacteria can use substrates (e.g., water-soluble sugars) released in the process (de Boer et al., 2005). In addition to fungi-derived C, root exudates are also the main C source for rhizosphere microbes (Philippot et al., 2013). Thus lower rhizosphere bacteria-fungi interactions in intercropping systems indicated an increased dependence on plant-derived nutrients, consistent with lower modularity in the rhizosphere (Figure 4B and Table 2). Therefore, intercropping potentially can affect the belowground ecological process of desert plants in arid environments.

Both bulk soil and rhizosphere *ps*ASVs from monoculture exhibited low-to-medium degrees of the co-occurrence networks (Figure 5), revealing that monoculture systems did not influence the highly co-occurring microbes. In contrast, intercropping systems significantly affected the highly co-occurring microbes, which possibly belong to hub microbiomes (microbial taxa that make more connections to their neighbors and play a disproportionately important role in structuring microbial communities) (Agler et al., 2016), revealing that these influential community members could be manipulated by the intercropping system. This hypothesis is further supported by the observation that *ps*ASVs also included highly abundant microbiome members, especially in the rhizosphere.

Keystone taxa (hub taxa with high abundance) are thought to be a key determinant of colonization for a wide range of occurring microbial taxa (Berry and Widder, 2014; Agler et al., 2016). In our study, all keystone ASVs of bulk soil and rhizosphere occurred in the intercropping system and were Actinobacteria-dominated (Figure 5 and Table 2), and the bulk soil community contained more keystone taxa than the rhizosphere (Table 2). Actinobacteria can produce a variety of bioactive substances, such as antibiotics, plant growth promoters, and enzymes, which can promote the growth of host plants and enhance their ability to resist environmental stress (Qin et al., 2011). These findings indicate that intercropping conducted in desert soil may introduce taxa to soil microbial communities by the functions of these keystone taxa. These keystone species possibly stem from endophytes and residues from other intercropping plants or are recruited by root exudates released from nearby plants (Chen et al., 2019).

It should be emphasized that the co-occurrence network visualizes correlations between microorganisms, including true ecological interactions (such as mutualism), but also non-random processes (such as niche overlap), and therefore does not necessarily reflect direct interactions between microorganisms (Faust and Raes, 2012; Weiss et al., 2016). Future experiments will evaluate whether these species identified as keystone or sensitive to planting patterns directly affect other members of the microbiome or indirectly affect other community members by affecting host performance and health (Agler et al., 2016; Hartman et al., 2017).

## Conclusion

Overall, the present study shows bacterial and fungal communities associated with *A. sparsifolia* and *K. caspica* differ between monoculture and intercropping systems, despite the lack of significant difference of bacterial richness. Plant genotypes play a limited effect on rhizosphere microbial communities in intercropping systems. Soil compartments did not differentiate fungal community in monoculture. Bacterial and fungal communities from bulk soil and rhizosphere respond differently to monoculture and intercropping systems. Comparing with bulk soil, the rhizosphere microbial community may be more resistant to environmental perturbation, and its overall complexity can be enhanced by intercropping. Our data also point out that intercropping systems can increase the modularity and stability of microbial co-occurrence networks in bulk soil and promote more microbial inter-kingdom interactions. Furthermore, microorganisms with different concurrence degrees have different responses to planting patterns. Monoculture systems did not influence the highly co-occurring microbes, while all these keystone taxa were presented in intercropping systems and were Actinobacteria-dominated. This paper provided a potential strategy to regulate soil microbial communities and rationally allocate plant species in desert ecosystems.

## Data Availability Statement

The datasets presented in this study can be found in online repositories. The names of the repository/repositories and accession number(s) can be found below: https://www.ncbi.nlm.nih.gov/, PRJNA750887.

## Author Contributions

ZZ: investigation, methodology, formal analysis, and writing-original draft. XC: investigation, resources, and data curation. AT: conceptualization, methodology, formal analysis, review and editing, and supervision. FZ: conceptualization, supervision, project administration, and funding acquisition. XL: review and editing. CG: methodology and review and editing. All authors contributed to the article and approved the submitted version.

## Conflict of Interest

The authors declare that the research was conducted in the absence of any commercial or financial relationships that could be construed as a potential conflict of interest.

## Publisher’s Note

All claims expressed in this article are solely those of the authors and do not necessarily represent those of their affiliated organizations, or those of the publisher, the editors and the reviewers. Any product that may be evaluated in this article, or claim that may be made by its manufacturer, is not guaranteed or endorsed by the publisher.

## Supplementary Material

Supplementary Data 1 Indicator species analysis.

Supplementary Data 2 *edgeR* analysis.

Supplementary Data 3 *ps*ASVs.

Supplementary Data 4 Keystone taxa.

Supplementary Data 5 Network analysis.

Supplementary Data 6 Taxonomic information.
