# Supplementary material for: Intercropping Systems Modify Desert Plant-Associated Microbial Communities and Weaken Host Effects in a Hyper-Arid Desert
Source: Front Microbiol. 2021 Nov 3;12:754453. doi: 10.3389/fmicb.2021.754453 (PMC8595258; doi:10.3389/fmicb.2021.754453)
Supplement: Supplementary Data 1 — Indicator species analysis. [file Data_Sheet_1.zip › Supplementary Figures.DOCX]

Supplementary Material

## Supplementary Tables

**Table S1** The soil properties of monoculture and intercropping system plots

| Soil  properties | Monoculture systems | | Intercropping  system |
| --- | --- | --- | --- |
|  | *Alhagi sparsifolia* | *Karelinia caspia* |  |
| DOC | 14.7±1.12 | 13.71±0.24 | 13.13±1.00 |
| SOM | 4.61±0.08 | 3.90±0.04 | 4.44±0.34 |
| TN | 0.235±0.001 | 0.188±0.002 | 0.223±0.014 |
| TP | 597.31±1.36b | 588.98±1.93a | 596.82±2.10a |
| TK | 17.65±0.03c | 18.03±0.03a | 17.82±0.02b |
| SWC | 0. 67±0. 09 | 1.15±0. 24 | 0. 75±0.02 |
| pH | 9.62±0.03 | 9.58±0.02 | 9.61±0.03 |
| EC | 100.02±5.44 | 122.6±11.67 | 95.9±8.95 |

**Note:** Different letters of each parameter indicate significant differences at *P* < 0.05 in different groups which can be divided into individual and interaction effects. Values are means ± SE (n=5).

*DOC* dissolved organic carbon (mg/kg), *SOM* soil organic matter (g/kg), *TN* total nitrogen (N) (g/kg), *TP* total phosphorus (P) (g/kg), *TK* total potassium (K) (g/kg), *SWC* soil water content (%), EC electric conductance (μs/cm).

**Table S2** Indicator species analysis on planting pattern-sensitive ASVs.

|  |  | Total ASVs |  | Sensitive ASVs | | |  |
| --- | --- | --- | --- | --- | --- | --- | --- |
|  |  |  |  | Total | Intercrop | Monoculture |  |
| Bulk soil | Bacteria | 3738 |  | 697 | 358 | 339 |  |
|  | Fungi | 261 |  | 36 | 24 | 12 |  |
| Rhizosphere | Bacteria | 2404 |  | 533 | 317 | 216 |  |
|  | Fungi | 174 |  | 35 | 25 | 10 |  |

Note: significance level (alpha): 0.05

## Supplementary Figures


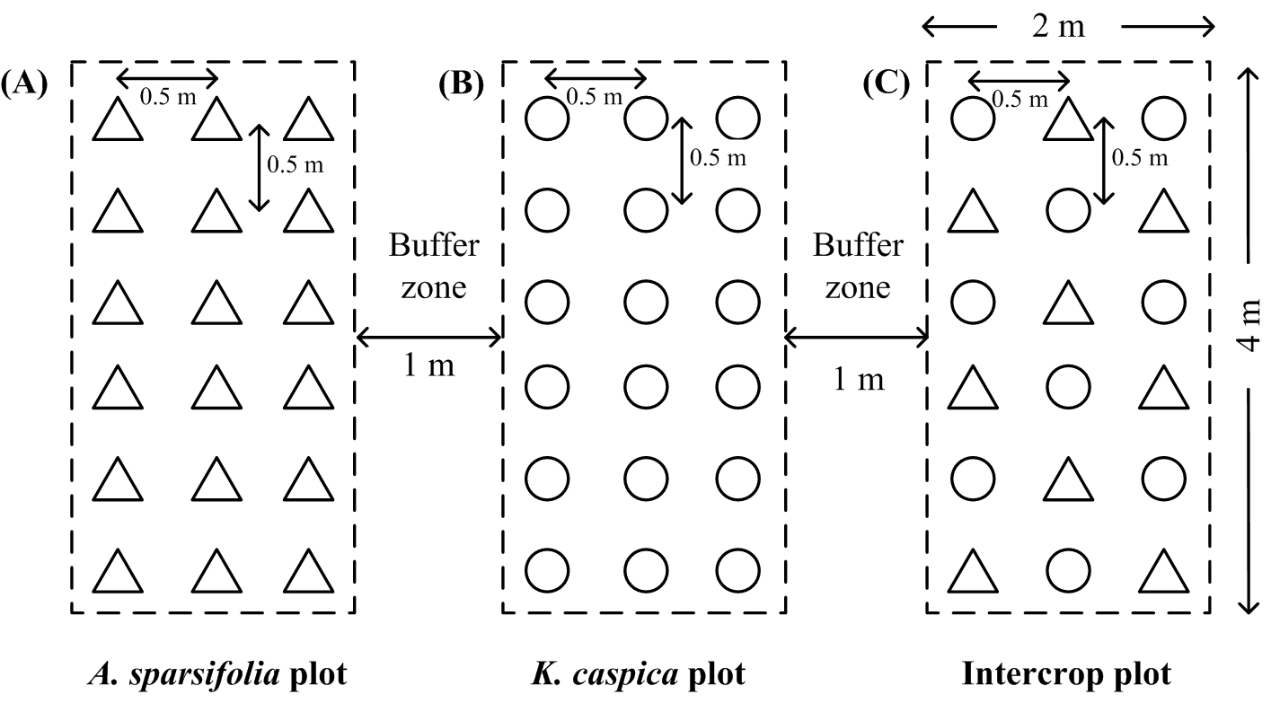


**Supplementary Figure 1.** Experimental design. Panels **(A)** and **(B)** show the monoculture plots of *A. sparsifolia* and *K. caspica*, respectively, and panel **(C)** shows their intercrop pattern. The triangle and circle represent the *A. sparsifolia* and *K. caspica*, respectively.


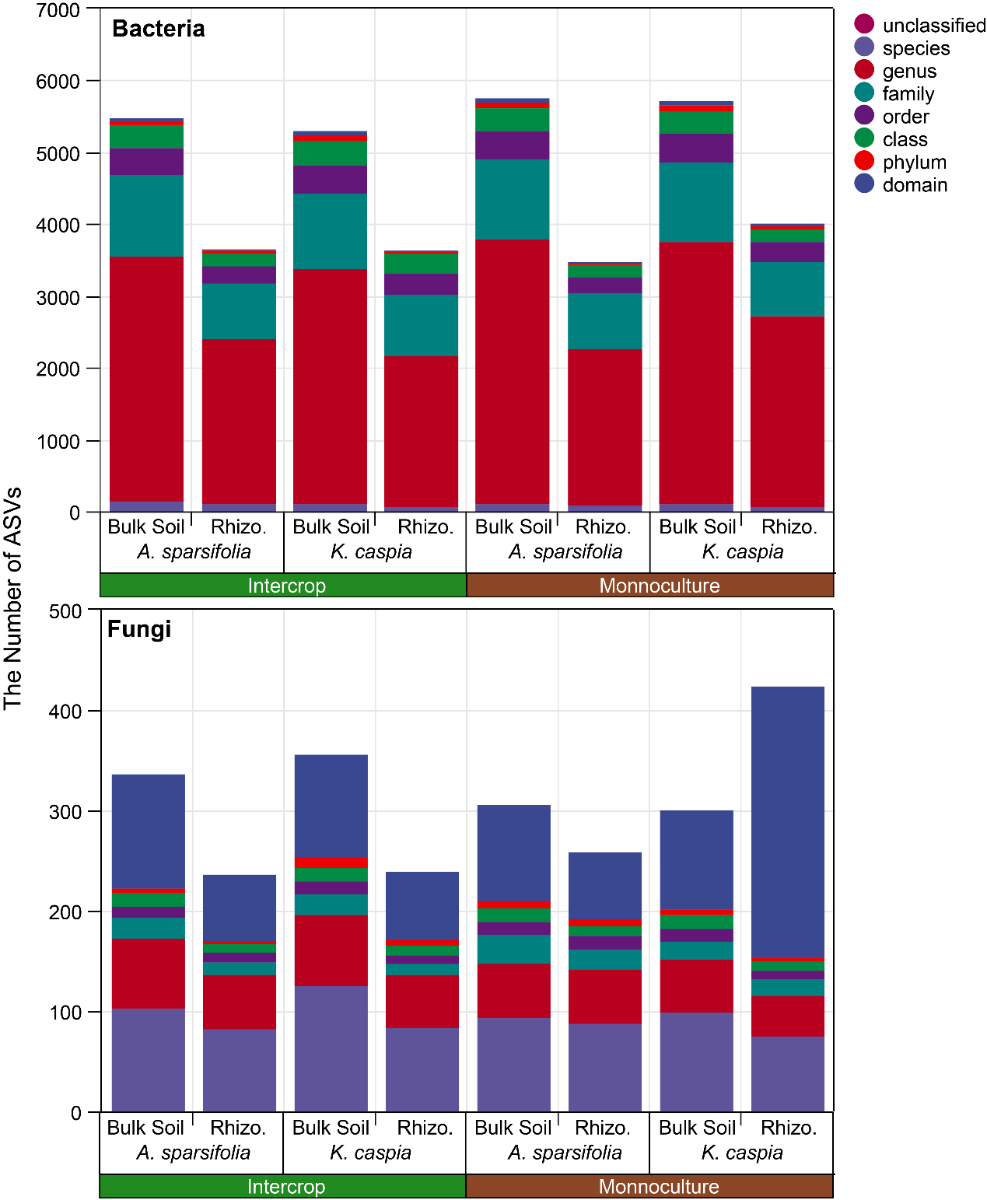


**Supplementary Figure 2.** The number of ASVs per sample. Y-axis represents the number of ASVs with the highest annotated classification level of the domain, phylum, class, order, family, genus, and species in annotation results (i.e., the number of ASVs only annotated these levels). Different levels of classification are indicated by different colors, and column heights correspond to the number of ASVs.


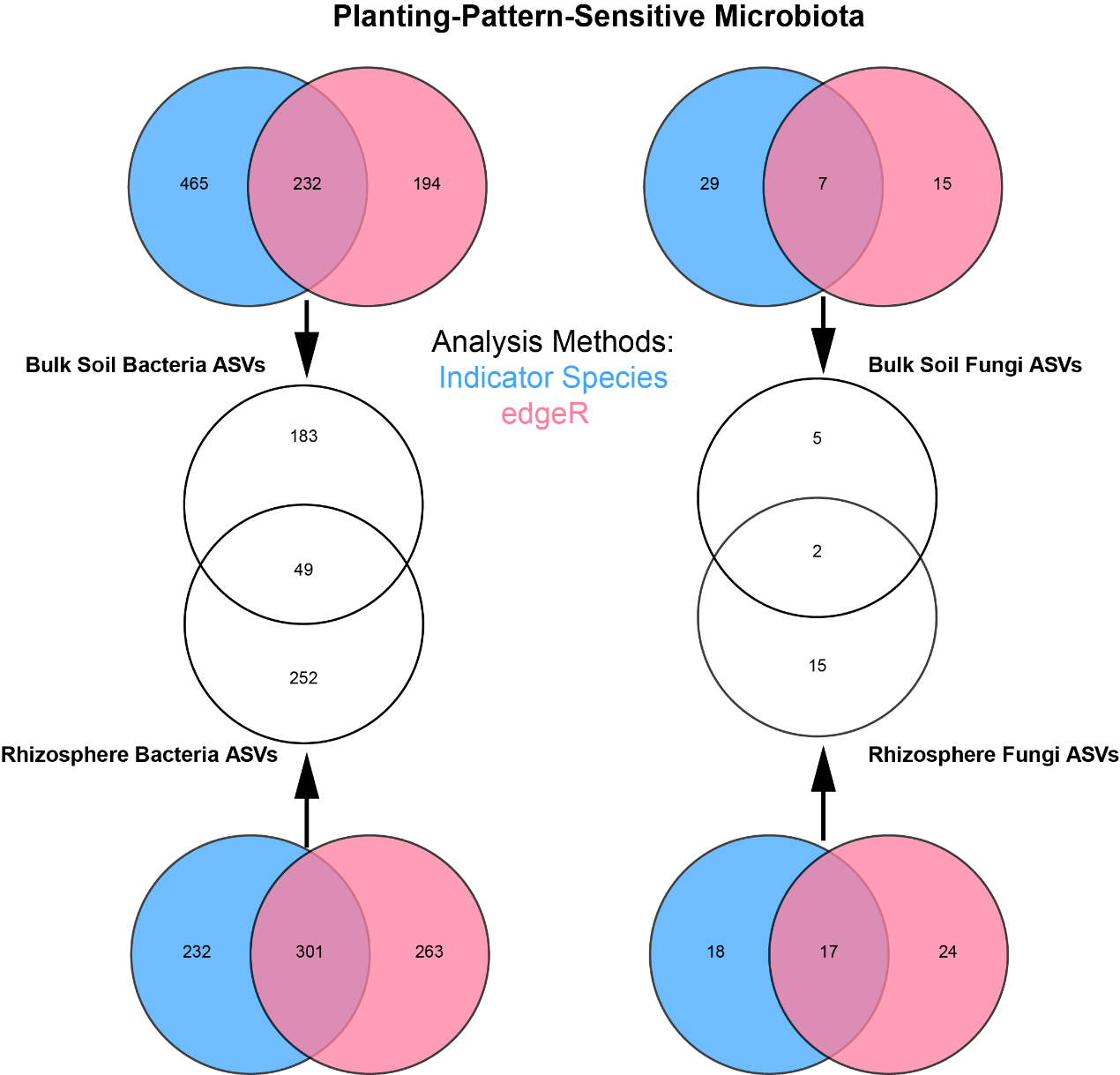


**Supplementary Figure 3.** The bacteria and fungi ASVs sensitive to planting modes in bulk soil and rhizosphere. Veen diagrams display the number of ASVs associated with planting modes identified with indicator species analysis (blue circle) and by edgeR (red circle). ASVs identified by both methods were defined as planting-pattern-sensitive ASVs. More information in Supplementary data1-2.


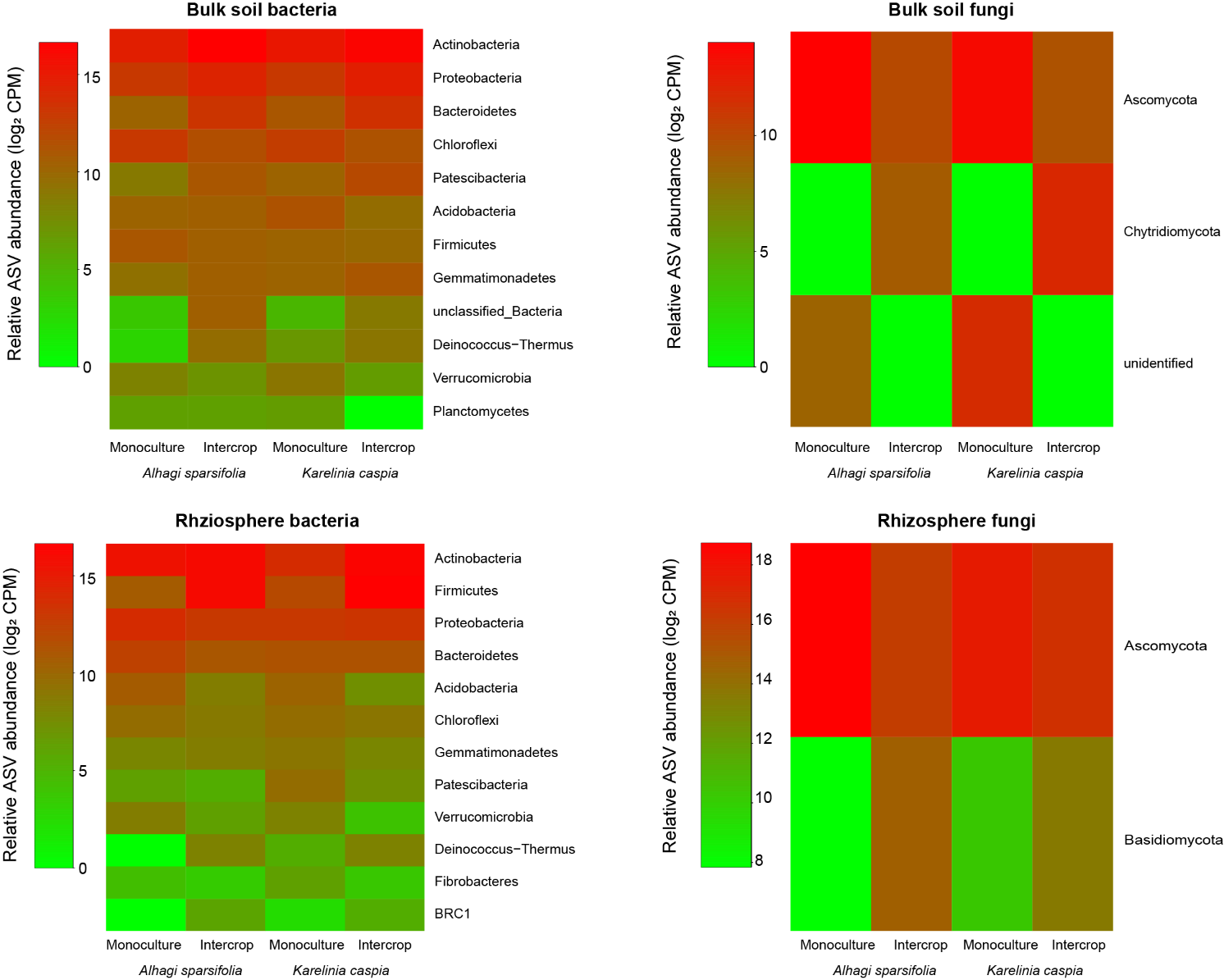


**Supplementary Figure 4.** The relative abundance (counts per million, CPM; log2 scale) of planting-pattern-sensitive ASVs identified from Fig. S4 at the phylum level.


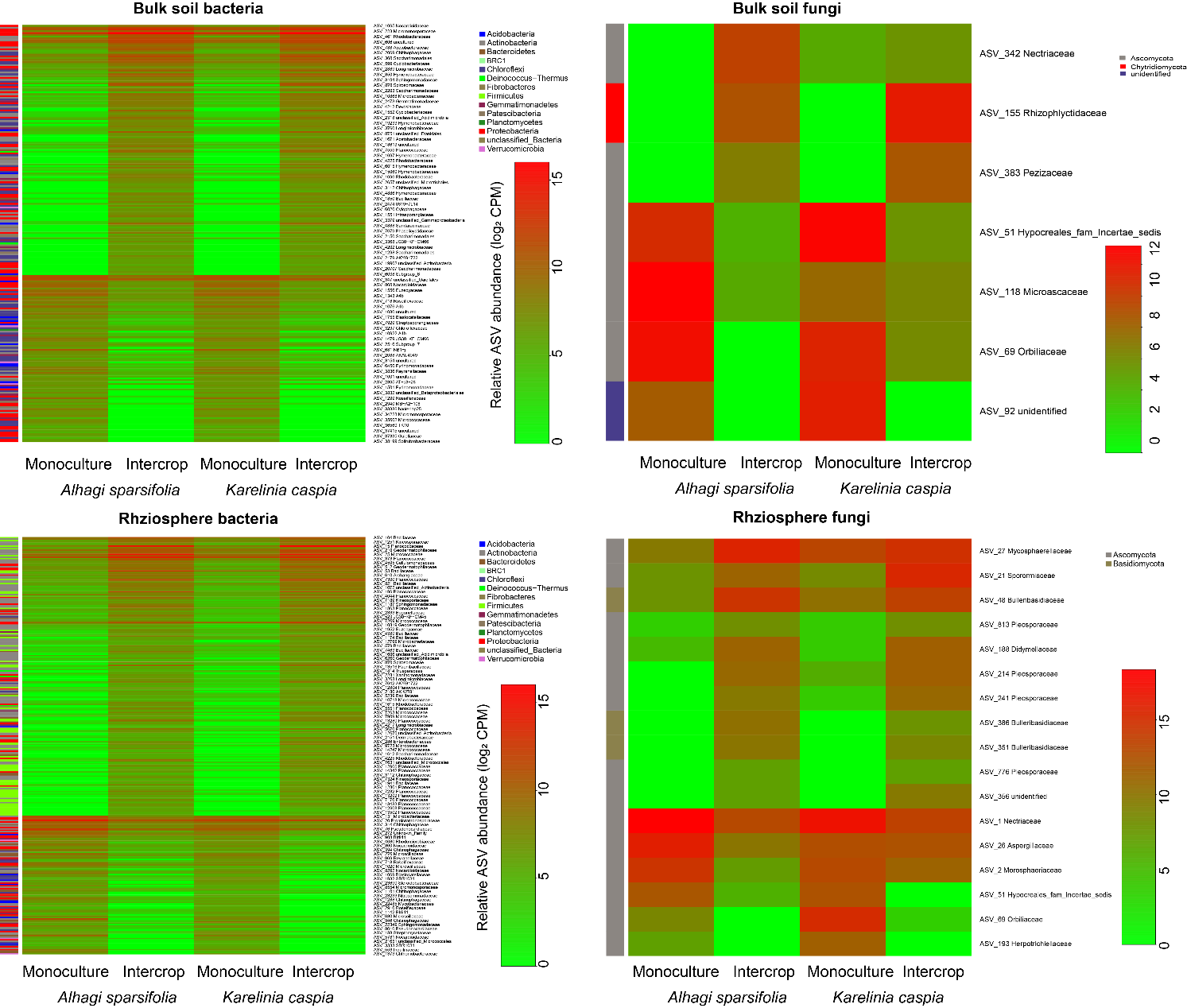


**Supplementary Figure 5.** The relative abundance (counts per million, CPM; log2 scale) of planting-pattern-sensitive ASVs identified from Fig. S4. ASVs are labeled with their family level taxonomy assignment, with the phylum level taxonomy assignment indicated by colored bars.


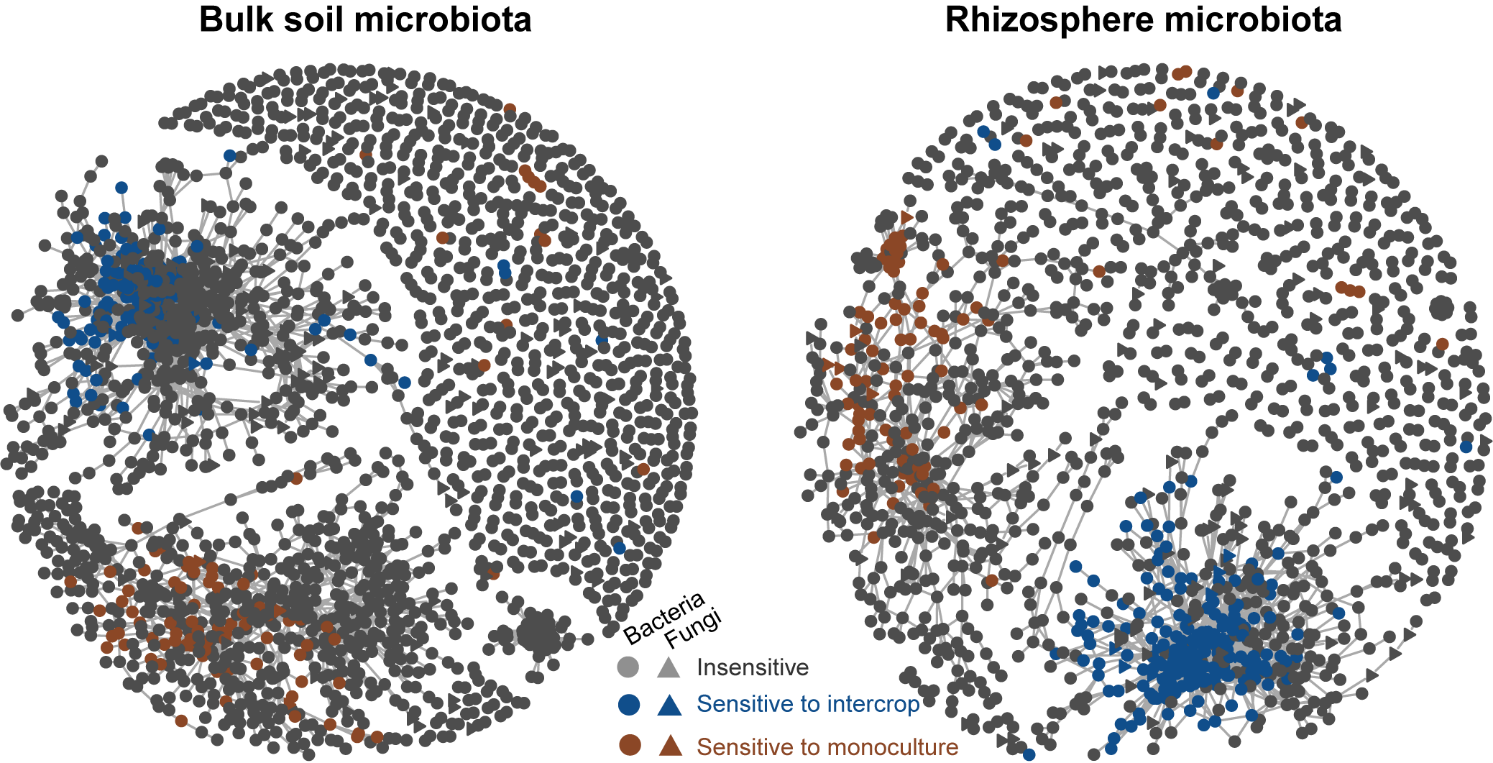


**Supplementary Figure 6.** Co-occurrence networks of microbial communities from bulk soil and rhizosphere in different planting patterns. The significant spearman’s correlations (ρ > 0.7, *P* < 0.001) between ASV pairs were visualized. The bacterial and fungal ASVs were represented by circles and triangles, respectively. The sensitive ASVs were colored by their association to the different planting patterns. The role and degree distribution of microorganisms are shown in Figure 5 More information are in Supplementary data 5.
